# Level of Pollution on Surrounding Environment from Landfill Aftercare

**DOI:** 10.3390/ijerph17062007

**Published:** 2020-03-18

**Authors:** Kristina Baziene, Ina Tetsman, Ramune Albrektiene

**Affiliations:** 1 College of Environmental Science & Engineering, Tongji University, Shanghai 200092, China; 2 Department of Mechanical and Material Engineering, Vilnius Gediminas Technical University, 10223 Vilnius, Lithuania; ina.tetsman@vgtu.lt; 3 Department of Chemistry and Bioengineering, Vilnius Gediminas Technical University, 10223 Vilnius, Lithuania; ramune.albrektiene@vgtu.lt

**Keywords:** landfill, soil pollution, surface water pollution, soil contamination factor, level of contamination

## Abstract

This work examines how the pollution from landfill aftercare effect the surrounding area and water basins. The subject of the study was a closed landfill where waste was disposed of without any accounting and operation of the landfill. During the study, soil, surface water, and sediment samples were taken over a two-year period. The data obtained compared with the maximum allowable concentrations established in the Northern part of Lithuania. The water sampling sites were selected taking into account the direction of the water flow, and the landfill was found to influence the water quality. Within 500 meters before the landfill, heavy metals and metalloid concentrations did not exceed the maximum allowable concentrations (Pb ≤ 20 µg/L; Ni ≤ 40 µg/L; Cr ≤ 100 µg/L; Cu ≤ 100 µg/L and As ≤ 20 µg/L). Soil and water sediment contamination factor and contamination level were determined for each metal and metalloid individually, which showed that as a single chemical element and its compounds none of them pose any danger to the environment. A different situation can be seen when calculating the total level of contamination, taking into account all pollutants classified as very hazardous, Z_d_ > 10. The results showed that monitoring (of surface water, including soil) and investigations, helping to reduce negative environmental impact, should be continued in the closed landfill.

## 1. Introduction

Waste management began to shift from a simple contamination control and treatment towards a more sustainable use and prevention approaches. Sustainable use had understood that ‘waste’ can become a ‘resource’ [1]. Following sustainable understanding of waste reuse, the final understanding of waste treatment technologies also had changed. Waste are used as recyclable materials help to prevent natural resources and minimize the effect on the environment, together affecting people health. All (not engineering designed) landfill in the European Union have been closed, by the requirements of EU [2,3,4,5]. Landfills generate and emit leachates, which formatting from the precipitation and waste humidity, and moves through the deposited wastes during the landfill operation and during landfill closing period. During the percolation processes different contaminants can be leached from landfill [6]. Residual pollution can be found in areas around landfill after the filling of the waste in dump side is stopped. The most aggressive pollutants are heavy metals and metalloids founded in landfill leachate [7,8]. Heavy metals and metalloids are the most stabile pollutants in the environment. Mostly all metals and metalloids with different amount are hazardous, which not only endangers for the environment but also affect human health [9]. Heavy metals and metalloids are washed from waste mass and accumulate in the environment, everywhere: in soils, surface water and in basin sediment. The greatest danger is the possibility that a metal through the food chain can get in the human body [10]. The accumulation of heavy metal and metalloid in soil severely affects the bioaccumulation processes [11,12,13,14,15]. Local people in different areas cultivate vegetables, grazing cattle a nearby landfill maintenance and are threatened by heavy metal and metalloids exposure. This work examines how pollution from closed landfills affect the closed territories and surface water pools. The heavy metals and metalloids are the pollutants in the environment [16,17,18], which can be found in soil and water and sediments. Five heavy metals and metalloid have found near landfill, which are arsenic (As), cadmium (Cd), copper (Cu), zinc (Zn) and lead (Pb) [19,20,21,22,23]. The same chemical elements, which may pose the greatest environmental and human health hazards have been researched in this study. The main aim of the research is to identify whether there is an environmental impact on surrounding area of landfill aftercare. The research done with three segments of environment: soil, water and sediments heavy metals and metalloid accumulation measurement and analysis.

## 2. Materials and Methods

**Figure 1:**
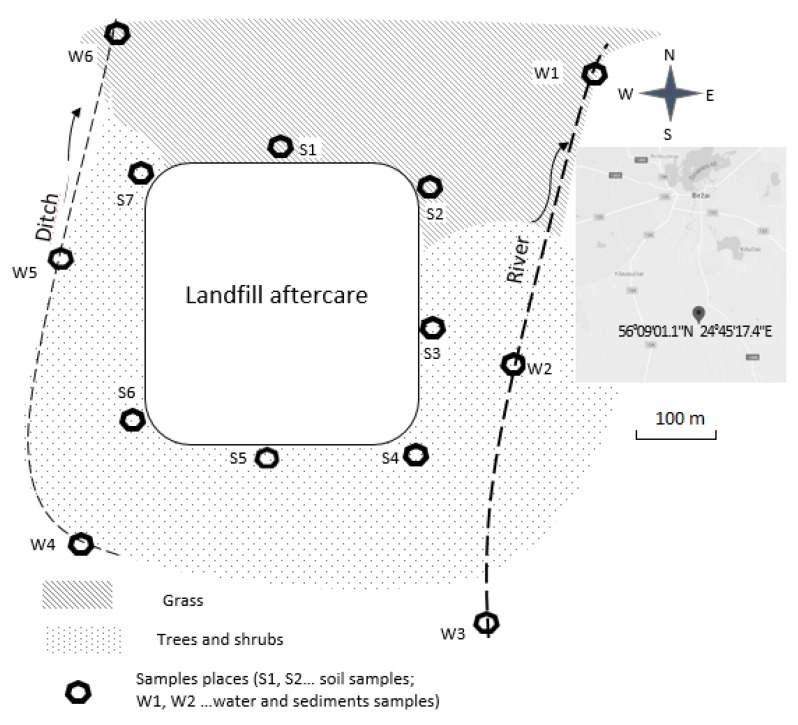
The scheme of sample points.

The research area has been chosen in the northern part of Lithuania. This type of landfill with the same composition of waste can be found in nearly every settlement in Lithuania. The landfill was located in cultivated fields and pasture environment. At present, the eastern, southern and western edge is near the forest, the north is coved by pastures. There is a small ditch in the west and a small river in the east. Both water pools are at a distance of about 200 meters from the landfill (Figure 1). The landfill has been in operation during 1986–2009. It is composed of health household, textile, plastic, wood, metal and other types of municipal waste. The apparent waste heap area is of about 3 hectares, the waste layer thickness of 5.0–8.0 m. In 2009, the landfill has been re-cultivated by spreading a thin surface layer (approximately 0.3 m), which is distributed very unevenly. There is no any leachate collection system in the landfill, that is why all precipitations percolate through the waste and filtrates in directly into the soil. During summer and autumn season there is >50% of rain, which can more leach pollution from the waste mass [24].

Water samples have been taken from both the ditch in the western part of the landfill and the river in the eastern part of the landfill. All samples have been taken three times (spring, summer and autumn season), when it is possible to take water samples (the river and the ditch usually are frozen during winter time), in two-year period. Thirty six water samples were randomly collected over a distance of 500 meter down stream and 500 meter up stream of the municipal landfill with glass bottle samplers. Twelve 1 liter bottle water samples were collected from the streams at the dump place. The glass bottles were washed and rinsed with deionized water. The water samples were taken, with the bottle’s mouth straight facing into the flow of the stream. All bottle samples have been labelled showing the sampling location and time. The samples were acidified with 10 mL of concentrated nitric acid, giving a pH < 2 to avoid adsorption of the metals on the glass bottlers walls. All samples were put into a cool box and taken to a laboratory for the heavy metal and metalloid analysis. The samples have been filtrated thought 0.45 µm cellulose acetate for the preparation of the measuring of the concentration of the metals. 50 mL of each samples prepared with deionized water in volumetric flasks was taken for metals concentration measure. Buck Scientific model 210 VGP (East Norwalk, Connecticut, USA) flame atomic absorption spectrometer was used for the measuring. The flame of acetylene (flow rate 2.0 L/min) and air (flow rate 13.5 L/min) was used for the measurement of samples. The concentration of metals was measured through injecting each sample (100 µL of prepared solution) through microinjecting system [25].

Soil samples have been taken from seven places around the landfill from different depths of the soil (S1, S2, S3, S4, S5, S6, S7). The waste pile is the abandoned waste while the surrounding soil is the immediate vicinity of the waste. Samples were collected at 0–15 cm, 15–30 cm, 30–45 cm, 45–60 cm depths from a circular area of approximately 15-cm diameter using manual corer marked. Afterwards, soil samples were carefully packed into plastic bags and taken for drying. Soil samples dried at up to 60 °C for 2 days [26]. Large debris, stones and plant remains were removed by hand picking while soil lumps were ground using porcelain mortar and pestle to achieve homogeneity. The ground soils were sieved through a 52 mm nylon sieve [27]. Samples were formed into pressed pellets using 8 g of sample and 0.4 g of binder [25]. The scientists [28,29] presented as X-ray absorption fine structure (EXAFS) spectroscopy is well suited to investigate metal speciation in soils, sediments. EXAFS spectra were extracted from raw X-ray absorption spectra from handheld XRF Analyzer. This method has detection limit as low as about 100 mg/kg for most heavy metals and usually it is suits and minimal sample preparation [30,31,32,33]. Equipment (Bruker’s S1 TITAN Handheld XRF Analyzer, Berlin, Germany) used for soil and sediments measurements.

The sediments samples were taken in the same places as water samples have been taken (W1, W2, W3, W4, W5, and W6), as shown in Figure 1. Every sample consists of approximately 300 g of surface layer sediment (0–3 cm). Polyethylene scoops were used for sampling and storage. Samples dried in an oven at <60 °C for 2 days. In order to normalize the variations in grain size distributions, the sediments samples were sieved through a 52 mm nylon sieve. The same method (X-ray absorption fine structure (EXAFS) spectroscopy) was used for measuring concentration of metals (As, Cr, Cu, Pb and Ni) in sediments samples.

The allowed assessment of heavy metal and metalloid soil contamination based on the ratio between the current content of the heavy metals and metalloid (in samples of soil) and the concentration of heavy metals and metalloid of a specific geochemical background (in current research the reference background was compared with measured data). Chemical analyses performed on three analytical samples taken from the 300 g composite sample of soil and sediments. The standard deviation values calculated using the data obtained from samples three replicates.

Soil contamination factor was used as analytic analysis of soil and sediments data. Soil contamination factor is the total concentrations of the metals for the evaluation of soil pollution [16,34]. The main indicator for soil contamination chemicals is the maximum level of hazardous chemicals in the soil. The higher the concentration of substances C (mg/kg) in the soil, the greater the hazard of soil contamination [35]. The concentration of substances in the soil comparing with maximum level of (MLC) the hazardous chemical concentration. The factor of contamination of the soil (K_0_) is determined of these substances, which expressed in terms of:

K_0_ = C/MLC (1)

C—measured chemical substances concentration in soil (mg/kg), MLC—chemical substance maximum level of concentration concentration in soil (mg/kg) [35].

The factor K_k_ of chemical element concentration is equal to:

K_k_ = C/C_f_ (2)

C_f_—the background content of the chemical element in the test soil sample (mg/kg) [35].

When soil is contaminated with more than one chemical substance or element, then soil contamination level is evaluated according to summed contamination coefficient Z_d_, following the formula:

Z_d_ = ∑ K_k_ − (n − 1) (3)

n—is number of chemical elements [35].

All calculated results of contamination coefficient Z_d_ were compared with Lithuania Hygiene Norm HN 60:2004 (maximum levels for hazardous substances in soil) Table 1.

## 3. Results and Discussion

### 3.1. Water Pollution

**Figure 2:**
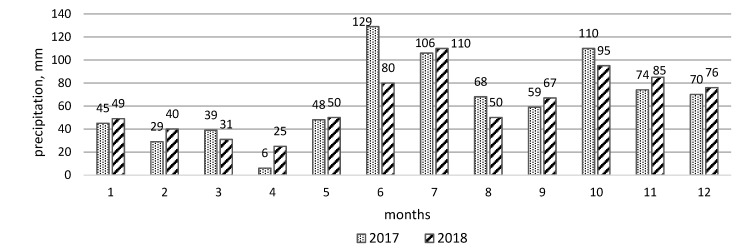
Varying quantities of precipitation in 2017 and 2018. (1—January, 2—February, … 12—December). [38,39].

**Figure 3:**
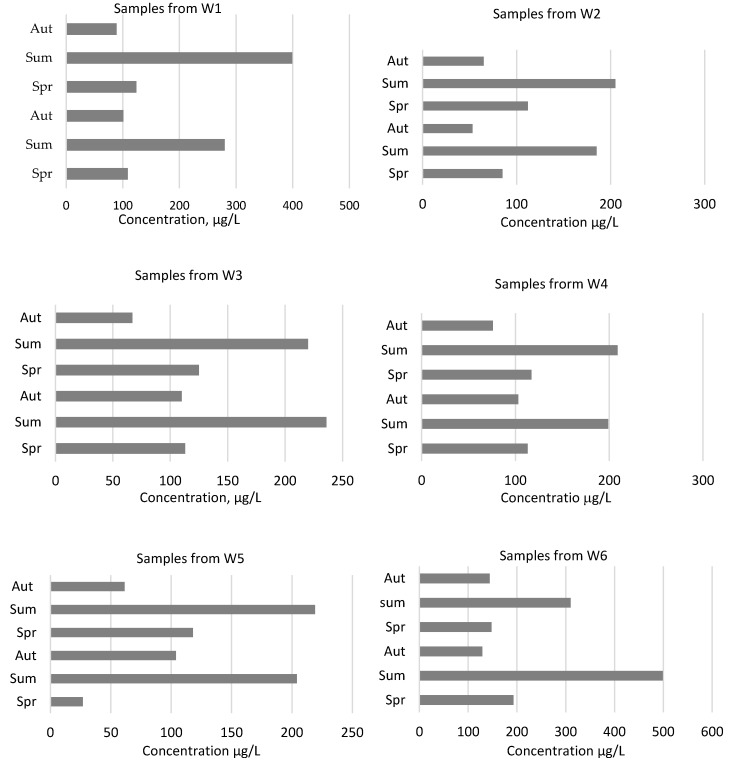
Total amount of contaminants in the river and ditch water during research period (Aut—autumn season, Sum—summer season, Spr—spring season).

The data reveal widespread pollution of both water pools (the ditch and river) as indicated by the presence of pollution of heavy metals and metalloid. The analysis of data from water samples can be done following three renaissance factors: seasons of year, pollutants and the place of sampling [36]. There are four seasons in Lithuania and the winter time is the one when usually the temperature is below 0 °C. The general amount of precipitation is about 690 mm per year. The biggest amount precipitates (average 230 mm [37]) from June to August, that is why the landfill after care can mostly be affected and the biggest effect of leaching of pollutants can be seen during this period (Figure 2). The amount of precipitation is varies from year to year, but the general tendencies are the same, that is why we can take an example of one year and look at the effect on the propagation increase of pollutants. Figure 3 shows, that during summer time the general concentration of compounds was the biggest and reached on average 220 µg/L during two years both in the river and the ditch. The changes of concentration during the whole year also shows, that spring has a bigger effect on water composition than autumn. Less than 50 µg/L number of compounds have been found in the spring time of 2018 and it was the result of smaller precipitation during the winter period.

During the research period of December 2017 and January and February 2018 there was small amount of precipitation in winter time, that is why the ditch have smaller effect of changes of composition of water (the ditch was less influenced by the changes of water composition). The scientists [40] in his study compared the characteristics of rainfall and found out that the pollution degree of Cu and Ni was proportional to the rainfall intensity. Rainfall is showing more effect than other factors such as soil permeability or type of vegetation. All water samples have shown, that during rain period the pollution of both river and ditch is more intense. Figure 3 shows, that effect on water concentration with different elements depends more on precipitation than on the pollution source (landfill). All sampling points at different places were mostly affected in summer time and the general concentration was from 200 µg/L to 500 µg/L during rain period (summer). 

The smallest concentration during summer measured at W3 and W4 points, which are before the landfill. The total amount of pollutants in water samples was less than 250 µg/L in summer period. Total pollutant concentrations over the next test period do not exceed 120 µg/L. 

**Figure 4:**
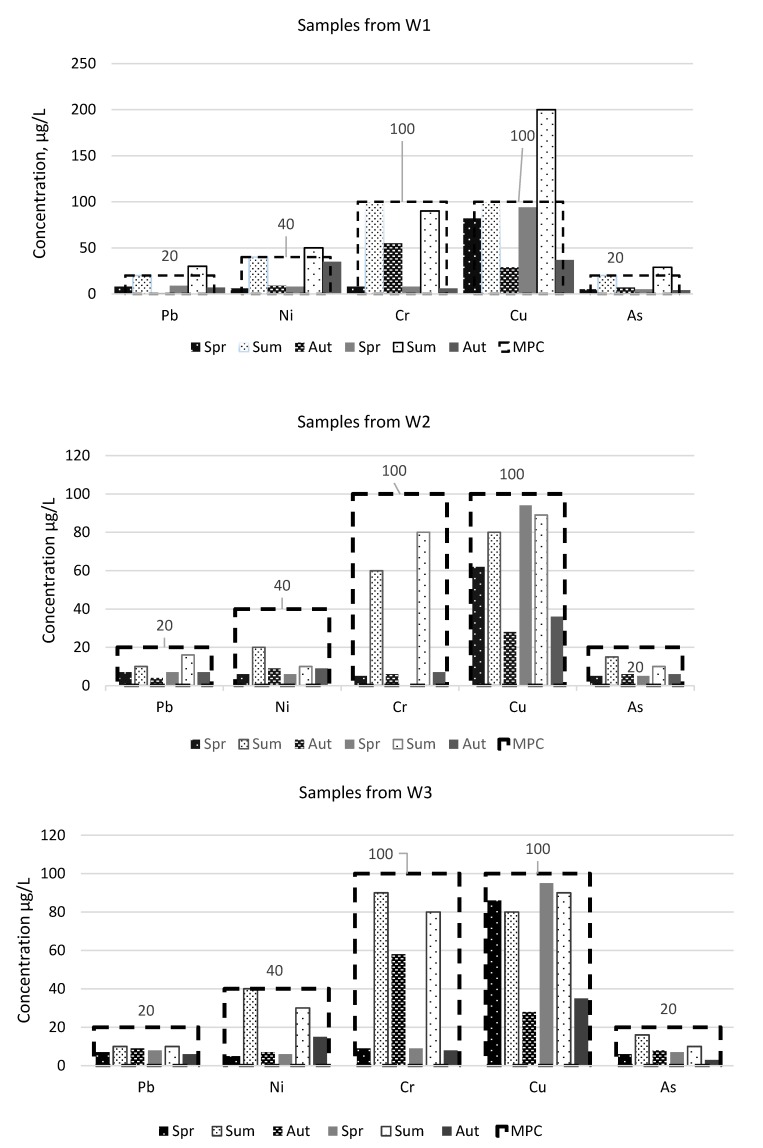
The concentration of heavy metals and metalloid in water of river during research period (Aut—autumn season, Sum—summer season, Spr—spring season, MPC—maximum permissible concentration for disposal treated wastewater, according to Waste Water Regulation of the Government of the Republic of Lithuania.

**Figure 5:**
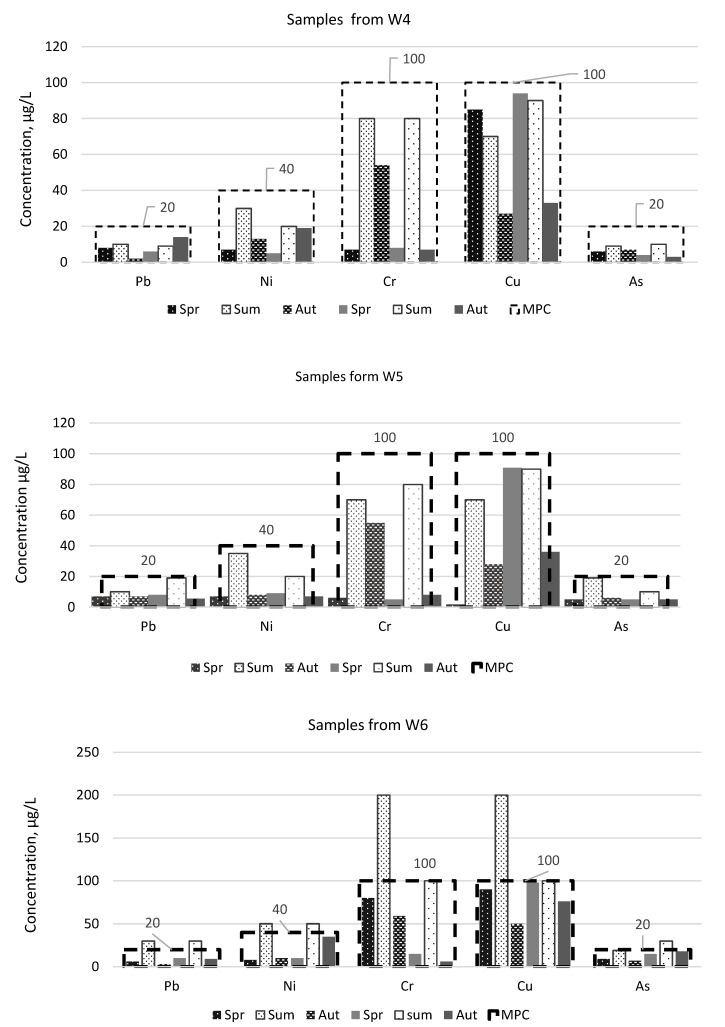
The concentration of heavy metals and metalloid in water of ditch during research period (Aut—autumn season, Sum—summer season, Spr—spring season, MPC—maximum permissible concentration for disposal treated wastewater, according to Waste Water Regulation of the Government of the Republic of Lithuania.

When analyzing the water contamination taking into account the sampling point, it is obvious that W2 and W5 points contain higher amounts of heavy metals and metalloid than the ones before the landfill. There is also a very significant increase in pollutants in the samples taken, respectively, at W1 and W6 points beyond the landfill. During the summer, the total amount of pollutants at the W1 and W6 sampling points was estimated at 400 and 600 µg/L, respectively. The difference in the total amount of pollutants indicates that a closed landfill will have an impact on adjacent water bodies, especially with increased rainfall. This shows that the bottom of the landfill is not insulated and that some of the pollutants, together with the ingress and absorption of precipitation, are leached into the surrounding environment and water bodies. During the analysis of the environmental impact of the closed landfill, the concentrations of lead, nickel, chromium, copper and arsenic in individual water samples taken were identified. The results obtained, compared against the allowable concentrations in Lithuania, are given in Figure 4 and Figure 5. By examining the individual quantities of each element the most prominent is the quantity of Cu, which exceeded the MPC two times in W1 sample. The quantity of Ni and As was also exceeded in the samples in the river water taken beyond the landfill. The concentrations of the heavy metals and metalloid in the sample water both before and at the landfill did not exceed the established maximum allowable levels. A similar situation is with the ditch water samples (Figure 5). An increase in contaminant concentrations is observed in the samples (W6) taken beyond the landfill. However, compared to the ditch water sample, it can be seen that the landfill has a greater impact on the ditch water than on the one from the river, as all elements researched had exceeded the maximum permissible concentrations: Pb, Ni, Cr, Cu and As—once during the period of two years, while lead and nickel—as much during two seasons. Chromium and copper concentrations were measured twice as high as the MPC, i.e., as 200 µg/L, during the first summer season. As those of the river water samples, the concentrations of the heavy metals and metalloids in ditch water samples from the areas before the landfill and at the landfill did not reach the MPC levels. [41], investigating heavy metal and metalloid levels in water, plant and animal samples, had also found out that the concentrations of Cr in the river beyond the landfill exceed the permissible ones. Although the above scientists did not investigate phonic concentrations, the increased levels of Cr are a consequence of the nearby landfill. Both the previous researches of scientists [22,42] and the present one had established that the greatest impact is observed in a landfill surrounds in the direction of water gradient. Water, as the best solvent, re-filters through the landfill, washes away the waste in a landfill together with the accumulated pollutant that enters a water body of together with the groundwater.

**Figure 6:**
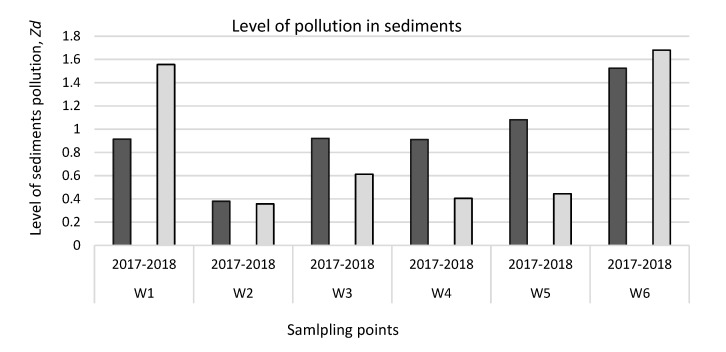
The level of pollution in sediments of river and ditch in 2017 and 2018 (W1, W2...W6—sampling points).

These results confirm that a closed landfill can have and does have a negative impact on the surrounding environment and especially on water bodies. When pollutants enter water bodies, they pollute the water itself and its ecosystems. Through the food chains, pollutants migrate into the aquatic flora and, eventually, into the aquatic fauna. During the metabolism cycle a large part of the living fauna and flora is converted into sediments of water bodies. Because heavy metals and metalloids tend to accumulate in organisms, accumulated pollutants end up as sediments of water bodies when organisms die and settle onto the bottom. Investigation of sedimentation of water bodies not only indicates the presence of pollution but also reveals the cumulative pollution. Concentrations of the four heavy metals and metalloids tested were determined for all the samples taken and the level of sediment pollution Zd was calculated together. The results obtained are shown in Figure 6. Level of pollution was calculated separately for each year, i.e., for 2017 and 2018. As shown in the diagram, the higher levels of pollution were found in 2017, except that more pronounced levels of pollution were found at points W1 and W6 in 2018. As W1 and W6 sampling sites are located behind the landfill in the direction of water flow, therefore the higher level of pollution and even pollution accumulation trend can be observed. There is also an increase in pollution at the landfill, but not as sharply as behind it. Compared to the established norms according to the Lithuanian Hygiene Standard HN 60:2004 [35], it can be seen that the pollution level at the landfill is less than 1 and it is classified as a safe pollution level, but still it is very close to the point where contamination is already assigned to the average hazard level. In W5 samples, the second level of contamination was detected in 2017. These results indicate that the closed landfill has an impact on the surrounding environment. This statement is also supported by other sediment samples behind the landfill, which is ranked second, being from 1.59 at W5 to 1.65 at W6. It was also observed that a greater effect was found in the ditch sediment. This level of pollution is influenced by different water flow rates and slower migration of pollutants together with the total mass of water. The higher levels of sediment pollution found in the ditch sediment indicate that the landfill has a greater impact on the ditch than on the river.

### 3.2. Soil and Sediments Pollution

Soil samples were also analyzed to determine the environmental impact of a closed landfill. During two years’ period soil samples were taken at different depths and heavy metal and metalloid concentrations were determined. As heavy metals and metalloids migrate in liquid media, the highest concentrations were found in deeper soil layers. It is important to note that according to the researches, the concentrations of heavy metals and metalloid found in the soil did not exceed the maximum allowable concentrations in Lithuania. Table 2 gives the estimated percentage of all measured pollutants in soil samples. 

Figures in indicate that concentrations exceed 70% of the maximum permissible level, but no concentrations exceeding the maximum levels have been found. The data presented shows that the highest amounts of heavy metals and metalloid are found in the deeper soil layers at a depth of 45–60 cm.

**Figure 7:**
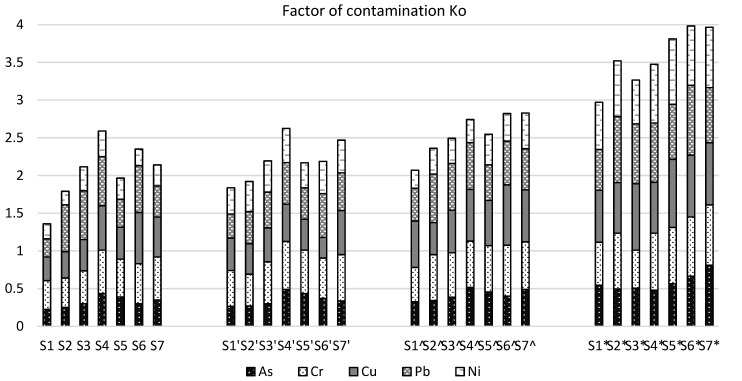
The factor of contamination Ko in different deep (S1, S2…S7—samples of 0–15 cm deep; S1’, S2’…S7’—samples of 15–30 cm deep; S1^, S2^…S7^—samples of 30–45 cm deep; S1*, S2*...S7*—samples of 45–60 cm deep).

Higher concentrations were also observed in 2017, indicating that pollutant migration is dependent on many factors such as rainfall, soil type, porosity of soil, type of grass cover, etc. When analysing the concentrations of the individual elements, it was observed that the maximum amounts were found for copper and lead, which suggests that waste was not identified in the closed landfill and the pollution with heavy metals and metalloid may continue to increase in the future as the degradation processes materials containing heavy metals and metalloids are slow and long lasting. The measurements also show that higher amounts of heavy metals and metalloid were found in the south-western part of the landfill, which suggests that it is in this part of the landfill where greater amounts of waste containing heavy metals and metalloid are buried. Also, one of the factors why the higher concentrations of pollutants were found in that part of a closed landfill may be uneven landfill cover and its thickness. Such conclusions could only be drawn from further research. When analyzing concentrations of each pollutant it is important to determine the contaminant factor. Figure 7 presents the estimated contamination factors for all samples at different depths for different metals and metalloids. The contamination factor allows a more detailed comparison of the results obtained uniformly, since the allowable concentrations of some metals and metalloids (e.g., of copper, lead) are much higher than, for example, arsenic or chromium. Figure 7 shows the distribution of the concentrations of heavy metals and metalloids. There is a tendency for the concentration to increase as the sampling depth increases: while the total contamination factor is less than 3 in the top layer, the total contamination with all heavy (metals and metalloid) is > 3 at the maximum depth (45–60 cm). 

**Figure 8:**
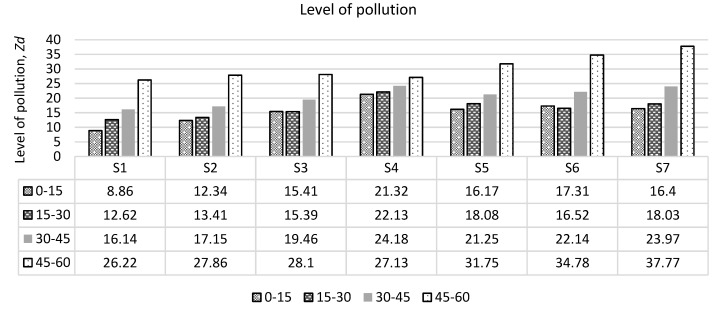
The level of pollution in sampling points of soil in different deep: 0–15 cm; 15–30 cm; 30–45 cm and 45–60 cm (S1, S2 ... S7—sampling points).

Contamination levels were calculated both in the research of water body sedimentation and soil samples. The estimated soil contamination levels are presented in Figure 8. The highest contamination factor and the level of contamination were found in the deepest layers (45–60 cm) collected in soil samples. Comparison of the results obtained with the levels determined in the hygiene standard (Lithuania HN 60:2004) [35] shows that only one point and only at the top layer (S1) has a hazardous level, whereas in other samples a very hazardous level is calculated, where Zd > 10. As the largest quantities of heavy metals and metalloid were found in the deepest layers, the level of contamination was highest in the layers at a depth of 45–60 cm. According to the distribution of points, the pollution is evenly distributed around the landfill and only the south-western part of the landfill has a higher level of contamination. [23]. There may be many reasons for this diffusion of heavy metals and metalloids, but this is mainly due to the composition of the waste being stored and the properties of the insulating material in the closed landfill.

The heavy metal and metalloid content of water, sediment and soil samples identified during the study indicate that the re-cultivated landfill should be continuously monitored, the data of such monitoring showing how much of this contaminated area poses a hazard to the environment. Such studies also show that even closed landfills have a long-term negative impact on the environment, which requires monitoring and assessment of whether there are new sources of contamination in the immediate vicinity of the landfill during long-term degradation processes.

## 4. Conclusions

The concentrations of four heavy metals and metalloid—(arsenic (As), cadmium (Cd), copper (Cu), zinc (Zn), and lead (Pb))—were determined in soil, surface water bodies and sediments of water bodies. Water and sediment analyses have shown that the closed landfill has an impact on the environment, as the increased pollutant concentrations had been identified in the direction of river flow. The concentrations of heavy metals (Cu, Cr, Pb and Ni) in the samples taken behind the landfill in the direction of the river flow exceeded the maximum allowable concentrations. The soil contamination coefficient determined during the investigations had shown that the area is not contaminated with each element individually, but when calculating the total contamination level, the results had shown that the area near the closed landfill is classified as very hazardous with Zd > 10. Heavy metal and metalloid migration from landfill to the environment is ongoing, but its intensity it is unclear. Long-term research is needed to determine the intensity of pollution and ways to reduce the negative impacts of the landfill. This increase in pollution indicates that the re-cultivated landfill needs to be monitored, especially for the surface water. Soil monitoring could also be included as one of the subjects of monitoring, since pollutants migrate through the soil and some part of the water, together with the pollutants, is used by plants and thus enters the ecosystem. Such dispersion of pollutants gives rise to a higher hazard to the environment surrounding the landfill.

## Figures and Tables

**Table 1:** Soil contamination on chemical substance evaluation [35].

**Soil Contamination Category**	**Z_d_**
Safe	Z_d_ < 1
Average hazard	1 < Z_d_ < 3
Hazard	3 < Z_d_ < 10
Very hazard	Z_d_ > 10

**Table 2:** The percentage of heavy metals and metalloid from the maximum level concentration in samples (MLC for soil (mg/kg): As—10; Cr—100; Cu—100; Pb—100; Ni—75) (HN 2004) [33].

				2017				2018			d
Soil Samples	Unit, Part of MLC	As	Cr	Cu	Pb	Ni	As	Cr	Cu	Pb	Ni
				**Samples from 0–15 cm deep**							
S1	%	20	38	26	20	16	25	38	37	28	24
S2	%	24	38	24	88	15	26	40	46	36	21
S3	%	31	42	46	**92**	27	29	45	37	37	37
S4	%	48	60	59	89	43	39	55	59	41	25
S5	%	39	55	41	48	28	39	45	44	26	28
S6	%	25	48	**80**	**87**	21	35	58	56	37	23
S7	%	32	58	67	59	27	38	56	39	23	29
				**Samples from 15–30 cm deep**							
S1	%	20	39	38	28	32	33	56	48	36	37
S2	%	29	38	32	40	45	25	46	49	45	35
S3	%	28	44	44	58	43	32	67	46	37	40
S4	%	56	60	47	65	57	42	67	52	45	34
S5	%	46	46	39	45	38	41	69	43	38	28
S6	%	37	48	50	67	47	37	59	55	49	38
S7	%	35	56	59	67	60	32	67	58	33	27
				**Samples from 30–45 cm deep**							
S1	%	30	35	55	40	16	35	56	68	47	32
S2	%	32	55	42	**76**	32	36	67	43	53	36
S3	%	46	56	64	**77**	27	31	62	49	47	40
S4	%	56	63	**79**	67	31	47	60	58	57	31
S5	%	52	54	56	46	45	39	69	64	48	36
S6	%	45	67	**87**	**78**	32	35	68	**73**	38	42
S7	%	52	67	69	57	60	46	59	69	52	35
				**Samples from 45–60 cm deep**							
S1	%	64	45	59	40	**89**	45	69	**79**	68	36
S2	%	44	**83**	48	**88**	**87**	55	65	**86**	**87**	61
S3	%	54	42	78	**92**	65	47	59	**98**	67	51
S4	%	48	**82**	67	**98**	**92**	48	69	68	59	64
S5	%	64	**79**	**98**	**78**	**97**	49	**70**	**83**	68	**76**
S6	%	**79**	**88**	**77**	**98**	**92**	54	69	**87**	**87**	66
S7	%	**97**	**89**	**87**	**78**	77	65	**71**	**78**	68	**83**
